# Reproductive risk factors of pterygium in postmenopausal women: a nationwide study in the Republic of Korea

**DOI:** 10.1038/s41598-021-91968-9

**Published:** 2021-06-14

**Authors:** Eunhae Shin, Dong Hui Lim, Tae-Young Chung, Gyule Han, Jung Eun Yoo, Keun Hye Jeon, Kyungdo Han, Dong Wook Shin

**Affiliations:** 1 Department of Ophthalmology, Samsung Medical Center, Sungkyunkwan University School of Medicine, Seoul, Republic of Korea; 2 Samsung Advanced Institute for Health Science and Technology, Sungkyunkwan University School of Medicine, Seoul, Republic of Korea; 3 Department of Family Medicine, Healthcare System Gangnam Center, Seoul National University Hospital, Seoul, Republic of Korea; 4 Department of Family Medicine, CHA Gumi Medical Center, CHA University, Gumi, Republic of Korea; 5 Department of Statistics and Actuarial Science, Soongsil University, Seoul, Republic of Korea; 6 Department of Family Medicine and Supportive Care Center, Samsung Medical Center, Sungkyunkwan University School of Medicine, Seoul, Republic of Korea; 7 Department of Digital Health, Samsung Advanced Institute for Health Sciences and Technology (SAIHST), Sungkyunkwan University, Seoul, Republic of Korea

**Keywords:** Diseases, Risk factors

## Abstract

This study is to elucidate the associations between female reproductive factors and pterygium. A total of 1,339,969 postmenopausal women in a retrospective cohort of Korean National Health Insurance Service data on ages 40 and above in 2009 was included. Cox proportional hazards regression was conducted to assess the hazard ratio (HR) for pterygium according to reproductive factors. Late menarche, early menopause, short reproductive period, increasing parity (≥ 2 children), breastfeeding (≥ 6 months), and no use of hormone replacement therapy (HRT) or oral contraceptive (OC) were significantly associated with risk of pterygium. In multivariate analysis, the HR for pterygium was 1.764 (95% confidence interval [CI], 1.529–2.035) for menarche age ≥ 17 years (reference: menarche age < 12 years). The HR of menopause age ≥ 55 years was 0.782 (95% CI, 0.724–0.845) (reference: menopause age < 40 years). The HR of parity ≥ 2 was 1.261 (95% CI, 1.148–1.385) (reference: nulliparity). The HR of breastfeeding ≥ 1 year was 1.663 (95% CI, 1.564–1.768) (reference: no breastfeeding). The HRs of HRT and OC use for any length of time were lower than those for the non-user groups (reference). Reproductive factors that increase estrogen exposure have protective effects against pterygium in females.

## Introduction

Pterygium is a triangular ‘wing-like’ growth consisting of conjunctival epithelium and hypertrophied subconjunctival connective tissue that occurs in the palpebral fissure and encroaches onto the cornea^1^. Previously known risk factors of pterygium include male sex, age, residence in a rural area, outdoor occupations, dry eye and ultraviolet (UV) light exposure^2–6^. Until recently, female sex had not drawn attention as risk factor of pterygium, since women are less prone to outdoor activities.

Regarding female, estrogen is a sex hormone thought to be a protective factor against several eye diseases in women ^7–9^. Recently, two articles covered estrogen effect on pterygium. Na et al. discussed the finding that postmenopausal estrogen therapy had a protective effect against flesh pterygium, and Pan et al. suggested that female sex acts as a protective factor for pterygium, but premature menopause increased the risk of pterygium in females^2,8^.

However, former studies on pterygium had a limited number of cases and did not include broad discussions on reproductive factors in females. In this retrospective cohort study with a large number of patient records from Korean National Health Insurance Service (NHIS) data, associations between pterygium and reproductive factors were revealed through multivariate analysis.

## Materials and method

### Data source and study setting

This is a nationwide retrospective cohort study to reveal the effect of estrogen on pterygium using the National Cancer Screening Program (NCSP) database by NHIS. The NHIS is the single insurer in Korea and provides a mandatory universal medical insurance system to 97% of the Korean population, while the Medical Aid program, financed by the government, covers the 3% of the population in the lowest income bracket. The NHIS databank contains databases compiling patient data pertaining to qualification (e.g., age, sex, income, region, and type of eligibility), claims (general information on specification, consultation statements, diagnosis statements defined by the International Classification of Disease 10th revision [ICD-10], and prescription statements), health check-ups (self-questionnaire on health behavior [e.g., past medical history, smoking, and drinking], anthropometric measurements [e.g., body mass index and blood pressure], and laboratory test results [e.g., fasting glucose and lipid levels]), and mortality^10–12^. And the NHIS initiated the NCSP in 1999, which includes biennial screening for breast cancer for all Korean women from the age of 40^13^.

This research followed the tenets of the Declaration of Helsinki. This study was approved by the Institutional Review Board of Samsung Medical Center (IRB File No. SMC 2019–07-045). The review board exempted the requirement for written informed consent due to the use of publicly available and anonymous data for analysis and the retrospective features of the study.

### Study population

**Figure 1:**
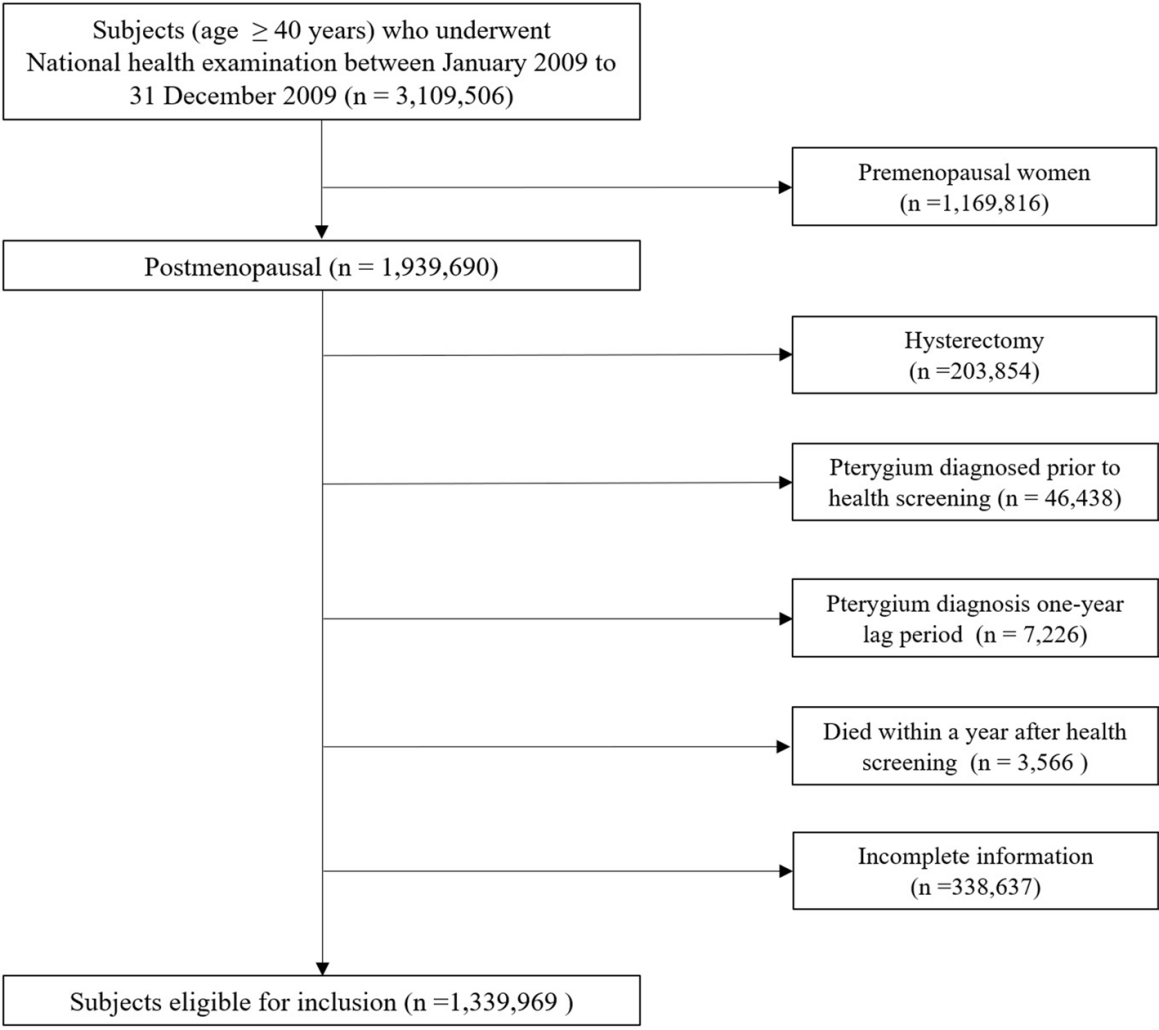
Flow chart of study population.

From the NHIS database, we collected data for women above the age of 40 who underwent breast cancer screening from 1 January 2009 to 31 December 2009. Among 3,109,506 female subjects, we identified 1,939,690 eligible postmenopausal women. We first excluded individuals who reported having a hysterectomy procedure (n = 203,854), as most did not know whether they underwent simultaneous oophorectomy. We defined pterygium with diagnostic codes from ICD-10. Individuals who had a diagnosis of pterygium before the health screening date (n = 46,438) were identified from the Korean NHIS medical service claims data and excluded. In addition, we required a one-year lag period and excluded individuals with a pterygium diagnosis within that year (n = 7,226). Individuals who died within one year after the health screening date were also excluded (n = 3,566). We excluded 338,637 individuals with missing data for at least one variable. A total of 1,339,969 individuals was included in the final analyses (Fig. 1).

### Reproductive factors

According to NCSP guidelines, the study subjects completed a questionnaire addressing their age at menarche, age at menopause, and parity. Information regarding total lifetime breastfeeding history, hormone replacement therapy (HRT) history, and use of oral contraceptives (OC) was collected. Age at menarche was categorized as ≤ 12 years, 13–14 years, 15–16 years, and ≥ 17 years, to be consistent with the distribution of age at menarche among Korean women. Age at menopause was categorized as < 40 years, 40–44 years, 45–49 years, 50–54 years, and ≥ 55 years. The reproductive period was calculated as the interval between the age at menarche and the age at menopause. Parity was categorized as 0, 1, or ≥ 2 children. Total lifetime breastfeeding history was categorized as never, < 6 months, 6–12 months, or ≥ 12 total months. The duration of HRT was categorized as never, < 2 years, 2–5 years, ≥ 5 years, or unknown. The duration of OC was categorized as never, < 1 year, ≥ 1 year, or unknown^10,14^.

### Study outcomes and follow-up

The endpoints of the study were pterygium diagnosis, which was defined with the following diagnostic codes defined by the Korean Standard Classification of Diseases 7th revision [KCD-7], with a few changes specific to Republic of Korea based on the International Classification of Diseases 10th revision: H11.0 (Pterygium), H11.01 (Central pterygium of the eye), H11.02 (Double pterygium of the eye), H11.03 (Peripheral pterygium of the eye), H11.05 (Recurrent pterygium of the eye), or H11.08 (Unspecified pterygium of the eye). The cohort was followed from one year after the health check-up date to the date of incident pterygium or until the end of the study period (December 31, 2018), whichever came first.

### Other variables

Several general factors regarding health status and variables regarding economic status were included in the statistical regression model. The selected factors were smoking, alcohol consumption, physical activity, body mass index, hypertension (HTN), diabetes mellitus (DM), dyslipidemia, income grade, and chronic kidney disease (CKD). Glomerular filtration rate (GFR, ml/min/1.73m^2^) was used as the criterion for CKD and divided into three categories.

The definitions of the lifestyle variables are as follows. Smoking status was categorized into three groups: non-smokers, current smokers who had smoked 100 cigarettes or more in their lifetime and is currently smoking or quitted for less than a month, and ex-smokers who had smoked 100 cigarettes or more in the past but had quit for at least one month^15,16^. Alcohol consumption status was categorized into three groups: non-drinkers, mild-drinkers who drank less than 30 g a day on average, and heavy-drinkers who drank more than 30 g a day. Regular exercise was defined as strenuous physical activity performed for at least 30 min at least five times a week.

Body mass index (BMI) was defined as weight in kilograms divided by the square of height in meters (kg/m^2^). BMI was divided into five categories: < 18.5 kg/m^2^, 18.5 to < 23 kg/m^2^ (normal), 23 to < 25 kg/m^2^, 25 to < 30 kg/m^2^ , and ≥ 30 kg/m^2^^14,17^. Waist circumference (WC) was analyzed to figure out metabolic aspects of postmenopausal women. Elevated WC was defined as ≥ 85 cm for women, according to the Korean Society for the study of Obesity's cut-off points for central or abdominal obesity^18^.

Subjects were considered to have HTN, DM, or dyslipidemia if they were on hypertensive, diabetic, or lipid-lowering medication, respectively. In addition, subjects with systolic blood pressure) ≥ 140 mmHg or diastolic blood pressure ≥ 90 mmHg, fasting blood glucose level ≥ 126 mg/dL, or triglyceride ≥ 240 mg/dL were considered to have HTN, DM, or dyslipidemia, respectively.

Household income status was classified into four groups in which Q1 and Q4 represent the lowest and highest income quartiles, respectively.

### Statistical analyses

Continuous variables are presented as mean ± standard deviation and median; interquartile range, and categorical variables are presented as number and percentage. Continuous variables were analyzed using Student t-test and categorial variables were analyzed using Chi-squared test in Table 1. Hazard ratio (HR) and 95% confidence interval (CI) values for pterygium were analyzed using the Cox proportional hazards model for various reproductive factors. The multivariate-adjusted proportional hazards model was applied: (1) Model 1 was not adjusted; (2) Model 2 was adjusted for age, age at menarche, age at menopause, parity, duration of breastfeeding, duration of HRT, duration of OC use, alcohol consumption, smoking, regular exercise, income, BMI, WC, HTN, DM, dyslipidemia, and CKD; and (3) Model 3 was adjusted for all variables as in Model 2, but age at menarche and age at menopause were replaced by reproductive period. Statistical analyses were performed using SAS version 9.4 (SAS Institute Inc., Cary, NC, USA), and a P-value < 0.05 was considered statistically significant. Multicollinearity among variables were analyzed by Variance Inflation Factor (VIF).

**Table 1:** Selected baseline characteristics of the study population.

	Total	Women diagnosed with pterygium	Women without pterygium	*p*-value*
n	1,339,969	37,157	1,302,812	
**Age** : Mean ± SD (median; IQR)	61.36 ± 8.28 (60; 54–68)	62.96 ± 7.6 (62; 57–68)	61.32 ± 8.3 (60; 54–68)	< .0001
**Age at menarche, No. (%)**				< .0001
Mean ± SD (median;IQR)	16.43 ± 1.83 (16; 15–18)	16.85 ± 1.81 (17; 16–18)	16.42 ± 1.83 (16; 15–18)	< .0001
≤ 12 y	13,052 (0.97)	190 (0.51)	12,862 (0.99)	
13–14 y	166,184 (12.4)	2,965 (7.98)	163,219 (12.53)	
15–16 y	525,658 (39.23)	12,752 (34.32)	512,906 (39.37)	
≥ 17 y	635,075 (47.39)	21,250 (57.19)	613,825 (47.12)	
**Age at menopause, No. (%)**				< .0001
Mean ± SD (median; IQR)	50.03 ± 4.01 (50; 48–52)	49.82 ± 4.14 (50; 48–52)	50.03 ± 4 (50; 48–52)	< .0001
< 40 y	22,685 (1.69)	780 (2.1)	21,905 (1.68)	
40–44 y	76,222 (5.69)	2,401 (6.46)	73,821 (5.67)	
45–49 y	364,484 (27.2)	10,075 (27.11)	354,409 (27.2)	
50–54 y	733,689 (54.75)	19,998 (53.82)	713,691 (54.78)	
≥ 55 y	142,889 (10.66)	3,903 (10.5)	138,986 (10.67)	
**Reproductive period, No. (%)**				< .0001
Mean ± SD (median; IQR)	33.6 ± 4.39 (34; 31–36)	32.97 ± 4.54 (33; 31–36)	33.61 ± 4.39 (34; 31–36)	< .0001
< 30 y	182,585 (13.63)	6,202 (16.69)	176,383 (13.54)	
30–34 y	558,560 (41.68)	16,770 (45.13)	541,790 (41.59)	
35–39 y	512,668 (38.26)	12,241 (32.94)	500,427 (38.41)	
≥ 40	86,156 (6.43)	1,944 (5.23)	84,212 (6.46)	
**Parity, No. (%)**				< .0001
Nulliparous	33,665 (2.51)	513 (1.38)	33,152 (2.54)	
1 child	82,930 (6.19)	1,239 (3.33)	81,691 (6.27)	
≥ 2 children	1,223,374 (91.3)	35,405 (95.28)	1,187,969 (91.18)	
**Duration of breastfeeding, No. (%)**				< .0001
Never	90,222 (6.73)	1,297 (3.49)	88,925 (6.83)	
< 0.5 y	89,576 (6.68)	1,336 (3.6)	88,240 (6.77)	
0.5 to < 1 y	235,749 (17.59)	4,933 (13.28)	230,816 (17.72)	
≥ 1 y	924,422 (68.99)	29,591 (79.64)	894,831 (68.68)	
**Hormone therapy, No. (%)**				< .0001
Never used	1,080,259 (80.62)	31,884 (85.81)	1,048,375 (80.47)	
< 2 y	123,101 (9.19)	2,478 (6.67)	120,623 (9.26)	
2 to < 5 y	50,800 (3.79)	894 (2.41)	49,906 (3.83)	
≥ 5 y	38,978 (2.91)	682 (1.84)	38,296 (2.94)	
Missing	46,831 (3.49)	1,219 (3.28)	45,612 (3.5)	
**Oral contraceptive use, No. (%)**				< .0001
Never used	1,069,278 (79.8)	30,393 (81.8)	1,038,885 (79.74)	
< 1 y	122,080 (9.11)	3,071 (8.26)	119,009 (9.13)	
≥ 1 y	81,920 (6.11)	1,988 (5.35)	79,932 (6.14)	
Missing	66,691 (4.98)	1,705 (4.59)	64,986 (4.99)	
**Smoking history, No. (%)**				< .0001
Never smoked	1,288,321 (96.15)	36,401 (97.97)	1,251,920 (96.09)	
Former smoker	14,613 (1.09)	228 (0.61)	14,385 (1.1)	
Current smoker	37,035 (2.76)	528 (1.42)	36,507 (2.8)	
**Alcohol consumption, No. (%)**				< .0001
None	1,170,472 (87.35)	33,200 (89.35)	1,137,272 (87.29)	
Mild	162,484 (12.13)	3,816 (10.27)	158,668 (12.18)	
Heavy	7,013 (0.52)	141 (0.38)	6,872 (0.53)	
**Regular physical activity, No. (%)**				< .0001
No	1,091,343 (81.45)	31,165 (83.87)	1,060,178 (81.38)	
Yes	248,626 (18.55)	5,992 (16.13)	242,634 (18.62)	
**Anthropometrics**				
**Systolic BP (mmHg)** : Mean ± SD (median; IQR)	125.54 ± 16.17 (125; 115–135)	126.93 ± 16.2 (127; 117–137)	125.5 ± 16.17 (125; 115–135)	< .0001
**Diastolic BP (mmHg)** : Mean ± SD (median; IQR)	76.85 ± 10.17 (78; 70–81)	77.75 ± 10.12 (80; 70–82)	76.83 ± 10.17 (78; 70–81)	< .0001
**Body mass index, No (%)**				< .0001
< 18.5 kg/m^2^	28,656 (2.14)	784 (2.11)	27,872 (2.14)	
18.5 to < 23 kg/m^2^	464,120 (34.64)	12,002 (32.3)	452,118 (34.7)	
23 to < 25 kg/m^2^	356,075 (26.57)	9,968 (26.83)	346,107 (26.57)	
25 to < 30 kg/m^2^	436,352 (32.56)	12,884 (34.67)	423,468 (32.5)	
≥ 30 kg/m^2^	54,766 (4.09)	1,519 (4.09)	53,247 (4.09)	
**Co-morbidity**				
**Hypertension, No (%)**				< .0001
No	728,659 (54.38)	19,025 (51.2)	709,634 (54.47)	
Yes	611,310 (45.62)	18,132 (48.8)	593,178 (45.53)	
**Diabetes Mellitus, No (%)**				0.0151
No	1,226,718 (91.55)	34,145 (91.89)	1,192,573 (91.54)	
Yes	113,251 (8.45)	3,012 (8.11)	110,239 (8.46)	
**Dyslipidemia, No (%)**				< .0001
No	880,686 (65.72)	25,082 (67.5)	855,604 (65.67)	
Yes	459,283 (34.28)	12,075 (32.5)	447,208 (34.33)	
**Laboratory findings**				
**Fasting glucose (mg/dl)** : Mean ± SD (median; IQR)	99.69 ± 24.22 (95; 87–105)	99.37 ± 23.6 (95; 87–104)	99.7 ± 24.23 (95; 87–105)	0.0101
**Total cholesterol (mg/dl) **: Mean ± SD (median; IQR)	208.22 ± 44.02 (206; 182–232)	207.55 ± 41.47 (205; 181–231)	208.24 ± 44.09 (206; 182–232)	0.003
**Glomerular filtration rate, No (%)**				0.0001
< 30	16,768 (1.25)	381 (1.03)	16,387 (1.26)	
< 60	138,775 (10.36)	3,776 (10.16)	134,999 (10.36)	
≥ 60	1,184,426 (88.39)	33,000 (88.81)	1,151,426 (88.38)	
**Income, No (%)**				< .0001
Q1 (lowest)	305,755 (22.82)	8,054 (21.68)	297,701 (22.85)	
Q2	249,733 (18.64)	7,062 (19.01)	242,671 (18.63)	
Q3	330,538 (24.67)	9,874 (26.57)	320,664 (24.61)	
Q4 (highest)	453,943 (33.88)	12,167 (32.74)	441,776 (33.91)	

Data are expressed as (mean ± standard deviation) and (median ± interquartile range) or n (%).

SD; standard deviation, IQR; interquartile range, BP; blood pressure.

*Student t-test for continuous variable, Chi-squared test for discrete variable.

## Results

### Baseline characteristics of the study population

The characteristics of the study participants are presented in Table 1. The mean age of the total population in this study was 61.36 ± 8.28 years (median; IQR = 60; 54–68). The overall mean ages at menarche and menopause were 16.43 ± 1.83 (16; 15–18) and 50.03 ± 4.01 (50; 48–52) years, respectively. Of these women, 91.3% had parity greater than 2, 68.99% had breastfed for more than 12 months, 80.62% were never HRT users, and 79.8% were never OC users.

Women with pterygium were older than women without pterygium (62.96 ± 7.6 [62; 57–68] and 61.32 ± 8.3 [60; 54–68] years, respectively), had a tendency to exercise less (16.13 vs. 18.62%), a tendency toward a higher BMI (over 25 kg/m^2^, 34.67 vs. 32.50%), and a higher prevalence of HTN (48.80 vs. 45.53%). Table 1 showed the characteristics of women diagnosed with pterygium and women without pterygium in detail. All factors listed in Table 1 showed a statistically significant difference between the two groups.

### Associations between reproductive factors and the risk of pterygium

Late menarche, early menopause, short reproductive period, increasing parity (≥ 2 children), breastfeeding (≥ 6 months), and no use of HRT or OC were significantly associated with risk of pterygium (Table 2).

**Table 2:** Hazard ratios (HR) and 95% confidence intervals (CI) for the association between reproductive factors and the risk of pterygium.

	Total	Pterygium cases	Follow-up Duration (person-years)	Incidence rate per 1000 person-years	HR(95% CI)		
					Model1	Model 2 (multivariable†)	Model 3 (multivariable‡)
**Age**					1.026 (1.025,1.027)	1.015 (1.013,1.016)	1.017 (1.015,1.018)
**Age at menarche**							
≤ 12 y	13,052	190	107,324.19	1.77034	1 (ref.)	1 (ref.)	
13–14 y	166,184	2,965	1,362,491.88	2.17616	1.229 (1.061,1.423)	1.121 (0.968,1.298)	
15- 16 y	525,658	12,752	4,279,835.54	2.97955	1.683 (1.458,1.942)	1.393 (1.207,1.608)	
≥ 17 y	635,075	21,250	5,127,957.11	4.14395	2.341 (2.029,2.7)	1.764 (1.529,2.035)	
p-for-trend					< .0001	< .0001	
**Age at menopause**							
< 40 y	22,685	780	181,384.75	4.30025	1 (ref.)	1 (ref.)	
40–44 y	76,222	2,401	611,940.54	3.92358	0.912 (0.841,0.989)	0.944 (0.87,1.023)	
45–49 y	364,484	10,075	2,959,407.54	3.4044	0.792 (0.736,0.852)	0.874 (0.813,0.94)	
50–54 y	733,689	19,998	5,962,296.86	3.35408	0.78 (0.726,0.838)	0.84 (0.782,0.902)	
≥ 55 y	142,889	3,903	1,162,579.03	3.35719	0.781 (0.723,0.843)	0.782 (0.724,0.845)	
p-for-trend					< .0001	< .0001	
**Reproductive period**							
< 30 y	182,585	6,202	1,464,824.51	4.23395	1 (ref.)		1 (ref.)
30–34 y	558,560	16,770	4,522,056.16	3.70849	0.876 (0.851,0.902)		0.917 (0.891,0.945)
35–39 y	512,668	12,241	4,188,131.53	2.92278	0.691 (0.67,0.712)		0.755 (0.732,0.778)
≥ 40 y	86,156	1,944	702,596.51	2.76688	0.654 (0.621,0.688)		0.683 (0.649,0.719)
p-for-trend					< .0001		< .0001
**Parity**							
Nulliparous	33,665	513	275,751.53	1.86037	1 (ref.)	1 (ref.)	1 (ref.)
1 child	82,930	1,239	680,681.93	1.82023	0.979 (0.883,1.085)	0.885 (0.796,0.984)	0.871 (0.783,0.969)
≥ 2 children	1,223,374	35,405	9,921,175.26	3.56863	1.918 (1.758,2.093)	1.261 (1.148,1.385)	1.249 (1.137,1.372)
p-for-trend					< .0001	< .0001	< .0001
**Duration of breastfeeding**							
Never	90,222	1,297	740,691.47	1.75107	1 (ref.)	1 (ref.)	1 (ref.)
< 0.5 y	89,576	1,336	736,523.22	1.81393	1.036 (0.96,1.118)	0.978 (0.903,1.059)	0.974 (0.9,1.054)
0.5 to < 1 y	235,749	4,933	1,928,992.47	2.55729	1.461 (1.374,1.553)	1.226 (1.149,1.309)	1.25 (1.17,1.334)
≥ 1 y	924,422	29,591	7,471,401.56	3.96057	2.262 (2.14,2.391)	1.663 (1.564,1.768)	1.73 (1.627,1.838)
p-for-trend					< .0001	< .0001	< .0001
**Hormone therapy**							
Never used	1,080,259	31,884	8,741,410.46	3.64747	1 (ref.)	1 (ref.)	1 (ref.)
< 2 y	123,101	2,478	1,015,766.33	2.43954	0.67 (0.643,0.698)	0.775 (0.744,0.808)	0.771 (0.74,0.804)
2 to < 5 y	50,800	894	419,556.55	2.13082	0.585 (0.548,0.626)	0.683 (0.639,0.73)	0.677 (0.633,0.724)
≥ 5 y	38,978	682	321,938.89	2.11841	0.582 (0.54,0.628)	0.647 (0.599,0.698)	0.638 (0.591,0.689)
Missing	46,831	1,219	378,936.48	3.2169	0.882 (0.833,0.934)	0.942 (0.886,1.002)	0.943 (0.887,1.003)
p-for-trend					< .0001	< .0001	< .0001
**Oral contraceptive use**							
Never used	1,069,278	30,393	8,668,104.56	3.5063	1 (ref.)	1 (ref.)	1 (ref.)
< 1 y	122,080	3,071	998,839.74	3.07457	0.878 (0.846,0.911)	0.923 (0.889,0.958)	0.928 (0.894,0.964)
≥ 1 y	81,920	1,988	669,788.21	2.9681	0.847 (0.81,0.887)	0.873 (0.834,0.913)	0.88 (0.841,0.921)
Missing	66,691	1,705	540,876.21	3.15229	0.899 (0.856,0.944)	0.978 (0.928,1.031)	0.968 (0.919,1.02)
p-for-trend					< .0001	0.0004	0.0006
**Smoking**							
Never smoker	1,288,321	36,401	10,464,020.01	3.47868	1 (ref.)	1 (ref.)	1 (ref.)
Former smoker	14,613	228	117,715.55	1.93687	0.556 (0.488,0.633)	0.655 (0.575,0.746)	0.646 (0.567,0.736)
Current smoker	37,035	528	295,873.16	1.78455	0.511 (0.469,0.557)	0.555 (0.509,0.605)	0.551 (0.505,0.601)
p-for-trend					< .0001	< .0001	< .0001
**Alcohol consumption**							
Non	1,170,472	33,200	9,488,458.05	3.49899	1 (ref.)	1 (ref.)	1 (ref.)
Mild (< 30 g/d)	162,484	3,816	1,331,833.65	2.86522	0.82 (0.793,0.848)	0.95 (0.919,0.983)	0.953 (0.921,0.986)
Heavy (≥ 30 g/d)	7,013	141	57,317.02	2.46	0.704 (0.597,0.831)	0.905 (0.767,1.069)	0.912 (0.772,1.076)
p-for-trend					< .0001	< .0001	0.0001
**Regular physical activity**							
No	1,091,343	31,165	8,840,724.99	3.52516	1 (ref.)	1 (ref.)	1 (ref.)
Yes	248,626	5,992	2,036,883.73	2.94175	0.836 (0.813,0.859)	0.891 (0.867,0.916)	0.897 (0.873,0.923)
**Body mass index (kg/m**^**2**^**)**							
< 18.5	28,656	784	222,498.63	3.52362	1.098 (1.022,1.18)	1.009 (0.938,1.085)	1.002 (0.932,1.077)
18.5 to < 23	464,120	12,002	3,762,054.76	3.19028	1 (ref.)	1 (ref.)	1 (ref.)
23 to < 25	356,075	9,968	2,899,278.15	3.4381	1.079 (1.05,1.108)	1.045 (1.017,1.074)	1.05 (1.022,1.078)
25 to < 30	436,352	12,884	3,549,624.04	3.62968	1.139 (1.111,1.167)	1.053 (1.023,1.084)	1.057 (1.027,1.088)
≥ 30	54,766	1,519	444,153.14	3.41999	1.072 (1.017,1.131)	0.98 (0.924,1.04)	0.978 (0.922,1.038)
p-for-trend					< .0001	< .0001	< .0001
**Waist circumference**							
< 85 cm	973,589	25,947	7,919,300	3.27643	1 (ref.)	1 (ref.)	1 (ref.)
≥ 85 cm	366,380	11,210	2,958,308	3.78933	1.156 (1.131,1.182)	1.036 (1.008,1.064)	1.036 (1.008,1.064)
**Co morbidity**							
**Hypertension**							
No	728,659	19,025	5,964,174.33	3.18988	1 (ref.)	1 (ref.)	1 (ref.)
Yes	611,310	18,132	4,913,434.38	3.69029	1.156 (1.132,1.179)	1.017 (0.994,1.039)	1.018 (0.996,1.041)
**Diabetes Mellitus**							
No	1,226,718	34,145	9,975,884.59	3.42275	1 (ref.)	1 (ref.)	1 (ref.)
Yes	113,251	3,012	901,724.12	3.34027	0.974 (0.938,1.011)	0.912 (0.878,0.947)	0.913 (0.879,0.948)
**Dyslipidemia**							
No	880,686	25,082	7,146,446.99	3.50972	1 (ref.)	1 (ref.)	1 (ref.)
Yes	459,283	12,075	3,731,161.73	3.23626	0.922 (0.902,0.942)	0.915 (0.895,0.936)	0.915 (0.895,0.936)
**Glomerular filtration rate**							
≥ 60	1,184,426	33,000	9,648,008.07	3.4204	1 (ref.)	1 (ref.)	1 (ref.)
30 to < 60	138,775	3,776	1,095,777.58	3.44595	1.003 (0.97,1.038)	0.868 (0.838,0.898)	0.861 (0.832,0.892)
< 30	16,768	381	133,823.08	2.84704	0.83 (0.75,0.918)	0.833 (0.753,0.922)	0.828 (0.748,0.916)
p-for-trend					0.0595	< .0001	< .0001
**Income**							
Q1 (lowest)	305,755	8,054	2,481,328.62	3.24584	1 (ref.)	1 (ref.)	1 (ref.)
Q2	249,733	7,062	2,030,237.22	3.47841	1.073 (1.039,1.107)	1.075 (1.041,1.11)	1.074 (1.04,1.109)
Q3	330,538	9,874	2,686,803.31	3.675	1.133 (1.101,1.167)	1.101 (1.069,1.133)	1.1 (1.068,1.133)
Q4 (highest)	453,943	12,167	3,679,239.56	3.30693	1.019 (0.991,1.048)	0.972 (0.945,1)	0.963 (0.936,0.99)
p-for-trend					0.152	0.0123	0.0006

IR, incidence rate; HR, hazard ratio; CI, confidence interval.

^†^ Adjusted for age, age at menarche and menopause, parity, duration of breastfeeding, duration of HRT, duration of oral contraceptive use, alcohol consumption, smoking, regular exercise, income, body mass index, waist circumference, hypertension, diabetes mellitus, dyslipidemia and glomerular filtration rate.

^‡^ Adjusted for age, reproductive period, parity, duration of breastfeeding, duration of HRT, duration of oral contraceptive use, alcohol consumption, smoking, regular exercise, income, body mass index, waist circumference, hypertension, diabetes mellitus, dyslipidemia and glomerular filtration rate.

In model 2, the HR for pterygium was 1.764 (95% CI, 1.529–2.035) in the oldest age at menarche group (≥ 17 years) compared to the youngest age at menarche group (≤ 12 years). The HR was 0.782 (95% CI, 0.724–0.845) in the oldest age at menopause group (≥ 55 years) compared to the youngest age at menopause group (< 40 years). The HR showed a dose–response relationship with age at menarche and age at menopause. In parity, HR significantly decreased to 0.885 (95% CI, 0.796–0.984) in the single parity group and increased to 1.261 (95% CI, 1.148–1.385) in the ≥ 2 parity group compared to the nulliparity group. The HR was increased to 1.226 (95% CI, 1.149–1.309) in the group that breastfed for 6–12 months and to 1.663 (95% CI, 1.564–1.768) in the group that breastfed for ≥ 12 months compared to that with no breastfeeding history. The HR decreased significantly in both HRT and OC users compared with never users.

Furthermore, when model 3 was adjusted for reproductive period instead of age at menarche and age at menopause as in model 2, similar trends were observed. The reproductive period was calculated as the interval between the age at menarche and the age at menopause (Table 2). The HR decreased significantly in proportion to reproductive period. The HR was 0.917 (95% CI, 0.891–0.945) in group of reproductive period 30–34 years, 0.755 (95% CI, 0.732–0.778) in group of reproductive period 35–38 years and 0.683 (95% CI, 0.649–0.719) in group of reproductive period ≥ 40 years (reference: reproductive period < 30 years).

### Other factors associated with pterygium

In model 2, the HR increased to 1.045 (95% CI, 1.017–1.074) in the group with BMI of 23 to 25 kg/m^2^ and to 1.053 (95% CI, 1.023–1.084) in the group with BMI of 25 to 30 kg/m^2^, compared to the normal BMI group. HR of group with WC ≥ 85 cm was 1.036 (95% CI, 1.008–1.064). Results of other covariate factors such as smoking, alcohol consumption, physical activity, HTN, DM, dyslipidemia, GFR, and income are shown in Table 2.

## Discussion

Our study demonstrated lifetime estrogen exposure as a protective effect against pterygium in females. In this nationwide cohort study investigating the association between pterygium and reproductive factors, adjusted multivariate analysis revealed statistically significant protective effect with factors of early age at menarche, later age at menopause, longer reproductive period, parity (single childbirth, reference: nulliparous), no breastfeeding, HRT, and OC use. Such associations were independent of possible confounding factors of smoking, alcohol consumption, regular physical activity, BMI, HTN, DM, dyslipidemia, GFR, and income. This study is the first nationwide population-based study on the effects of reproductive factors on pterygium in the Republic of Korea.

The associations between reproductive factors and risk of pterygium have been discussed in the few literature. HRT in postmenopausal women lowered the prevalence of fresh pterygium, and premature menopause increased the risk of grade 2 or higher pterygium in females^2,8^. Such findings correspond with our results. However, previous researchs were conducted with limited population and revealed the effects of only few female reproductive factors. In this study, we had largest study cohort regarding this subject and showed effects of multiple female reproductive factors on pterygium. Early menopause, late menarche, longer reproductive period, single childbirth, shorter breastfeeding period, HRT, and OC use had a protective effect against pterygium in females.

Clues that lead to mechanism of estrogen on pteygium could be found in literatures. Previous studies have shown the presence of sex steroid hormone receptors (SSHR) in various ocular tissues, such as the lens, retina, choroid, cornea, iris, ciliary body, lacrimal gland, meibomian gland, lid, and palpebral and bulbar conjunctiva^19,20^. Estrogen, which combines with SSHR, has various functions. First, estrogen has been shown to decrease oxidative stress in tissues by preserving mitochondrial function, cell viability, and ATP level^21,22^. Oxidative stress caused by increased production of radical oxygen species (ROS) is thought to be the main pathogenesis of pterygium. Studies on immunohistochemical analysis showed staining of the oxidative stress marker 8-hydroxy-2'-deoxyguanosine in pterygium compared to normal conjunctival tissue^23,24^. Also, pterygium tissue had increased level of nitric oxide, increased total antioxidant status, and decreased antioxidant enzymes^25^. This indicates that high estrogen level prevents development of pterygium by reducing oxidative stress.

Furthermore, estrogen provides cell resistance to transforming growth factor β (TGF β) and promotes normal function of the cell membrane^9^. Growth factors such as TGF β show strong immunohistochemical staining in pterygium tissue and play an important role in cell proliferation, inflammation, connective tissue remodeling, and angiogenesis^26,27^. Estrogen reduces the risk of pterygium by hindering the action of growth factors.

Considering previous research and the results of our study, estrogen exposure, either exogenous or endogenous, seems to have antioxidative effects and plays a protective role against pterygium. Such protective effect of lifetime estrogen exposure on systemic diseases had been discussed in the literatures before. Several studies argued that more lifetime estrogen exposure in postmenopausal women was associated with lower mortality, less cardiovascular disease, and better cognitive function in later life^28,29^.

On the other hand, the association may not be causal relationship, as genetic or environmental factors could also determine lifetime estrogen exposure. There are some evidences that onset of age at menopause is affected by genetic factors, as evidenced by different menopausal age among races, associated gene loci^30^, and association between family history and premature menopause^31^. Moreover, environmental factors such as smoking, lower education, low occupation levels and lower family income are associated with premature or early menopause^31–33^. Therefore, there is a possibility that genetic or environmental factors determined the level of estrogen exposure and pterygium development. Also, estrogen might act as surrogator or mediator of genetic and environmental factors in pterygium development.

While single childbirth decreased the HR of pterygium, multiple births increased the HR. This seemingly opposite result should be interpreted with caution, considering the physiological plausibility. It is well known that estrogen level increases dramatically during pregnancy^34^. However, estradiol level decreases in consecutive pregnancies^35,36^. Moreover, in lactating women, prolactin level is increased and results in hypo-estrogenization^37^. A multiparous group would have a longer total lactation period compared to a single childbirth group, resulting in a longer hypo-estrogenization period. These results could explain the increased HR of pterygium in the multiparous group. Furthermore, VIF was analyzed to verify multicollinearity among variables statistically, and none of the variables showed VIF more than 10 to presume multicollinearity.

A preceding study on BMI and pterygium revealed that obesity in females had a significant association with pterygium in multivariate analysis^38^. This supports our result of increased HR in the group with BMI of 23 to 30 kg/m^2^. The study indicated that obesity is a chronic inflammatory disease and responsible for overproduction of ROS and nitrogen species, which cause oxidative stress^38^. Such oxidative stress is a well-known cause of pterygium.

The strength of our study lies in the use of a large population based on a national database. Traditionally, female sex was left out of the discussion on risk factors of pterygium and male sex was emphasized connected with environmental factors. This article focused solely on effects of female reproductive factors on pterygium. Secondly, unlike previous articles that suggested only a few reproductive factors to be associated with risk of pterygium, our study revealed that overall reproductive factors that increase estrogen level have a protective effect against the disease.

There are several limitations to our study. First, we could not obtain information on ultraviolet light exposure and dry eye in NHIS data, although these factors are usually included in a discussion of risk of pterygium^2,3,6,39^. Second, all the information on reproductive factors was based on self-reported histories; therefore, the possibility of bias caused by inaccurate recall cannot be excluded. Third, we were unable to retrieve more detailed data regarding age of initial HRT and OC use, the duration between menopause and HRT or OC initiation, the composition and concentration of HRT and OC (e.g., estrogen alone or combined estrogen-progesterone) and the dose, as these questions were not included in the questionnaires. Further detailed information about female hormone use and user potential risk factors for pterygium would be needed to clarify this possible association between exogenous female hormone use and risk of pterygium in future study. Lastly, as Korea has a homogenous ethnic population, further studies on other ethnic groups should follow.

In conclusion, based on a nationwide large population cohort, this study revealed that reproductive factors that increase female exposure to estrogen had a protective effect against pterygium.
